# An age stratified analysis of the biomarkers in patients with colorectal cancer

**DOI:** 10.1038/s41598-021-01850-x

**Published:** 2021-11-17

**Authors:** Hui Yao, Chengjie Li, Xiaodong Tan

**Affiliations:** 1 School of Public Health, Wuhan University, No. 115 of Donghu Road, Wuchang District, Wuhan, 430000 China; 2 Department of Neurology and Suzhou Clinical Research Center of Neurological Disease, The Second Affiliated Hospital of Soochow University, Suzhou, 215000 China

**Keywords:** Cancer, Molecular medicine

## Abstract

Colorectal cancer (CRC), a common malignant tumor of the digestive tract, has a high incidence and mortality rate. Several recent studies have found that aging is associated with the increasing risk of cancer. Nevertheless, the expression status and function of age-related genes in CRC is still not well understood. In the study, we comprehensively analyzed the gene expression data of CRC patients from The Cancer Genome Atlas (TCGA) database. Age-related differential expression genes (age-related DEGs) in tumor tissues compared with normal tissues of CRC were further identified. Gene Ontology (GO) and Kyoto Encyclopedia of Genes and Genomes (KEGG) enrichment analyses of age-related DEGs were performed by clusterProfiler of R. Afterwards, we used the STRING database to map the protein–protein interaction network of DEGs. We constructed prognostic model through univariate and multivariate COX regression analyses, and further evaluated their predictive power. The prognostic gene signature-related functional pathways were explored by gene set enrichment analysis (GSEA). The weighted gene co-expression network analysis (WGCNA) was used to identify key module associated with two prognostic gene signatures. Finally, we used the Metascape to perform functional enrichment analysis of genes in the key module. A total of 279 age-related DEGs were identified from the TCGA database. GO and KEGG enrichment analysis showed that the age-related DEGs were enriched in the Modulation of chemical synaptic transmission and Neuroactive ligand–receptor interaction. Moreover, we established a novel age-related gene signature (DLX2 and PCOLCE2) for overall survival in CRC, which was further predicted in both the training and validation sets. The results of GSEA demonstrated that numerous disease-related pathways were enriched in the high-risk group. We identified 43 genes related to the DLX2 and PCOLCE2 by the WGCNA co-expression network. We also found that these 43 genes were enriched in the cancer-related pathways. To sum up, the study identified an age-related gene signature for predicting the prognosis of CRC patients, which is conducive to the identification of novel prognostic molecular markers.

## Introduction

CRC, which covers colon and rectal cancers, is the third most prevalent cancer worldwide and the fourth leading cause of death from cancer^1–3^. Although the overall incidence of CRC is declining, especially in high-income countries, the morbidity of young populations has shown an upward trend. The incidence of CRC was reported to have declined by about 3% per year between 2003 and 2012, but increased by 2% in those under 50 years of age^4,5^. The prediction results from the Surveillance, Epidemiology, and End Results (SEER) database indicate that by 2030 the incidence of colon cancer will increase by 90.0% and 27.7% for patients 20–34 years and 35–49 years, and rectal cancer will increase by 124.2% and 46.0%, respectively^6^. The concept of early-onset colorectal cancer has emerged in the context of the younger age of onset of CRC. Some researchers define early-onset colorectal cancer as CRC in people under the age of 50 and applied it in specific studies, although the widely recognized definition of this concept needs further efforts^7,8^. Finally, a recent large population study showed that a higher polygenic risk score was actually more strongly associated with early-onset CRC development at a cut-off age of 50 years of diagnosis, especially if there was no family history of colorectal cancer^8^. Therefore, we speculate that 50 years old is a meaningful cut-off value for the occurrence, metastasis and prognosis of colorectal cancer, and try to take 50 years old as the basis for grouping.

Age is a widespread adverse factor in the occurrence of cancer^9^. Previous studies have shown differences in histological features and prognosis between elderly and young patients with cancer^10,11^. These findings emphasize the importance of diagnostic age on prognosis, but the impact of age on different tumors and the role of age-related genes in clinical decision-making need to be further explored. Currently, we are concerned about the dramatic and unexplained upsurge in the incidence of CRC among the younger people^12^. The available evidence suggests that young CRC patients exhibit different disease staging, histological characteristics, and molecular characteristics variation variants than older ones, and that patients with early-onset CRC present with unique genetic characteristics^13,14^. A previous large population study showed that patients with colorectal cancer under the age of 50 had fewer BRAF V600 mutations than those over the age of 50, which means special consideration and further investigations should be performed^15^. The identification of prognostic biomarkers is known to be important in guiding patients to systemic therapy for optimal therapeutic benefit^16,17^. However, the development of prognostic biomarkers for CRC and the evaluation of their predictive role are currently limited^18,19^. Meanwhile, it is not yet clear whether age-related biomarkers can be applied as prognostic markers in patients with CRC. Therefore, it is crucial to analyze the prognostic value and mechanisms of age-related genes in the CRC.

In this study, we obtained the transcriptome data and corresponding clinical information of CRC patients from TCGA database, and analyzed the differential expression of age-related genes between normal and CRC patients to explore their potential functions. We further constructed and validated a prognostic model based on differentially expressed age-related genes, and analyzed the biological functions associated with the prognostic signature by GSEA. Finally, genes associated with prognostic signatures were screened by WGCNA, and the key genes. The results of this study provide biomarkers for prognosis in patients with colorectal cancer.

## Material and methods

### Data processing

We searched the transcriptome data and corresponding clinical data of 41 normal samples and 460 CRC samples from the public database TCGA (https://portal.gdc.cancer.gov/). The database platform have obtained ethical approval of participants and all methods were performed in accordance with the relevant guidelines and regulations. The 460 patients with CRC were divided into old (equal or older than 50 years) and young (younger than 50 years) groups according to the age at initial pathologic diagnosis. There were 407 CRC patients in the old group and 53 CRC patients in the young group (Supplementary Table S1). A total of 444 cases of CRC were selected for subsequent analysis by excluding that the patients who had no survival time or survival status.

### Identification of differentially expressed genes (DEGs)

The DEGs between normal and CRC samples or young group and old group were analyzed using R software packages ‘edgeR’, respectively. The cutoff criteria were |log_2_ fold change (FC)|> 1 and *P* value < 0.05. The visualized volcano maps of genes were done using the ‘ggplot2’ of the R package.

### GO and KEGG pathway analysis of DEGs

The biological functions of these 279 DEGs were comprehensively detected by GO enrichment and KEGG pathway analysis. The GO analysis terms included cellular component (CC), molecular function (MF), and biological process (BP). All enrichment analyses were carried out by utilizing the ‘Cluster Profiler’ package of R software. A *P* value less than 0.05 was considered as statistically significant.

### Protein–protein interaction (PPI) network construction

The STRING database (https://string-db.org/) was used to construct a PPI network of 279 DEGs. Then, the data were imported into the software of Cytoscape for visual presentation.

### Construction and verification of the prognostic signature

The 444 cases of CRC were randomly grouped into a training set (n = 310) and a validation set (n = 134) according to a ratio of 7:3. Univariate Cox regression analysis was performed to filtrate the selected overall survival (OS)-related DEGs in the training set. Then, the multivariable Cox regression analysis was carried out to establish the prognosis model based on the DEGs associated with OS. The risk score calculating formula was:

$${\text{Risk score }} = {\text{ ExpGene1}}{*}{\text{Coef1 }} + {\text{ ExpGene2}}{*}{\text{Coef2 }} + {\text{ ExpGene3}}{*}{\text{Coef3}} \ldots$$

where Coef means the regression coefficients of genes, Exp is the normalized expression values of each gene signature. The formula was used to calculate the risk score for each CRC patient in the TCGA database. The median risk score was used as the threshold, based on which the CRC patient in the training set was divided into a high-risk group and a low-risk group. The survival differences between the two groups were calculated using the Kaplan–Meier (K–M) and log-rank test. In addition, a ROC curve was plotted to assess the 3- and 5-year survival probability of CRC patients using the ‘survivalROC’ R package. In the validation set, the same procedure was performed to validate the risk model. Further, the independent prognostic value of the risk model was evaluated using univariate and multivariate Cox regression analyses. These analyses were performed with the ‘survival’ and ‘survminer’ R packages. The R package ‘rms’ was used to establish a nomogram to predict the probability of 1-, 3-, and 5-year survival of BUC patients. The performance of the nomogram was evaluated using calibration curves.

### Gene set enrichment analysis (GSEA)

To inspect the different signaling pathways between the low- and high-risk groups, GSEA (version 4.1.0) was conducted by the ‘clusterProfiler’ package in R. The significantly enriched pathways in the two risk groups were selected by *P* value < 0.05.

### Construction of WGCNA

The R package ‘WGCNA’ was used to construct the co-expression network and to identify the co-expressed modules with prognostic genes signature. After excluding genes with an average expression amount less than 1, and the remaining genes were used to construct a co-expression network. The expression of DLX2, PCOLCE2, risk, and risk score was considered as clinical traits in the co-expression network. The power of β = 7 (scale-free R^2^ = 0.9) were selected as the soft-thresholding parameter to ensure a scale-free network, and the dynamic hybrid cut method was used to identify co-expressed gene modules.

### Statistical analysis

All analyses were performed using the R programming language. The Metascape was used to perform functional enrichment analysis of 43 key modular genes. In all analyses, *P* values less than 0.05 were considered as statistically significant.

### Ethics approval and consent to participate

Data was collected from public data repositories.

## Results

### Identification of age-related DEGs in CRC patients

**Figure 1:**
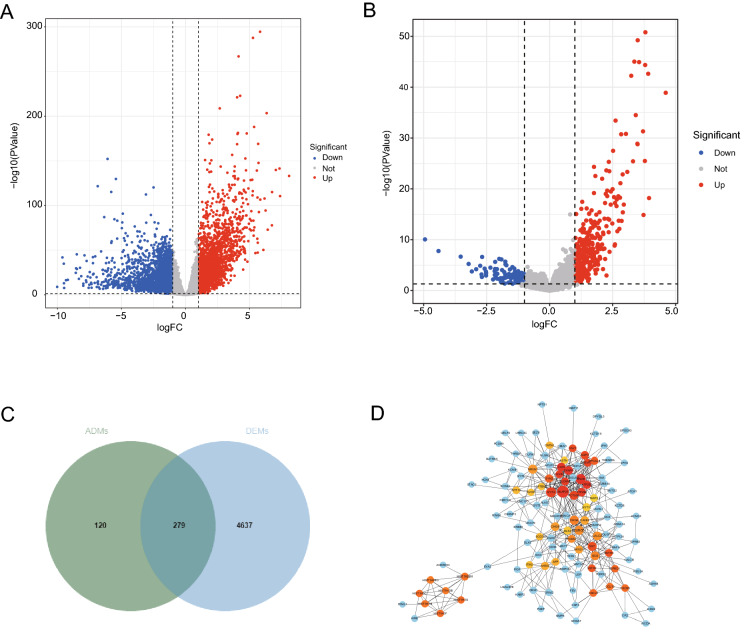
Differentially expressed genes in the TCGA database. (**A**) The DEGs between CRC and normal samples. (**B**) The DEGs between young and old groups. (**C**) The Venn diagram describes 279 common differentially expressed genes. (**D**) PPI network of 279 common genes. (software: The STRING database, version number: 11.0, URL link: https://version-11-0.string-db.org/).

To screen out the hub age-related genes that contribute to CRC malignant progression, we assessed the DEGs between CRC and normal samples. A total of 4916 DEGs were obtained from the TCGA database, including 2344 upregulated genes and 2572 downregulated genes (Fig. 1A and Supplementary Table S2). Next, we identified 399 DEGs between young and old groups, of which 65 DEGs were downregulated and 334 were upregulated (Fig. 1B and Supplementary Table S3). To reveal the DEGs associated with age, a Venn diagram was constructed (Fig. 1C), displaying 279 age-related DEGs that were overlapped between the 4916 DEGs and 399 age-related genes (Supplementary Table S4). These include genes such as SYT4, HPSE2 and KCNB1. To analyze the interrelationship among age-related DEGs, we constructed a PPI network. The top fifty genes with the strongest interactions were labeled red and yellow as shown in Fig. 1D and Supplementary Table S5. SNAP25 and SYT4 were hub DEGs with high degree of interactions in the PPI network.

### Functional enrichment analysis of DEGs

**Figure 2:**
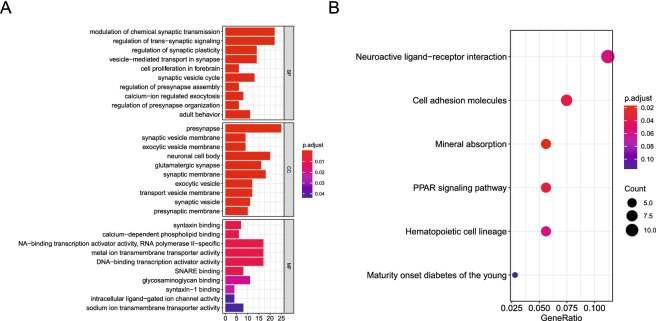
Functional enrichment analysis of DEGs. (**A**) The top 10 GO terms. (**B**) The top 10 KEGG pathways.

To investigate the potential regulatory mechanism of 279 age-related DEGs in CRC, we performed GO and KEGG enrichment analysis. Using the criteria of *P* value < 0.05, 86 BP terms, 26 CC terms, 11 MF terms and 6 KEGG terms were enriched in our study. The top 10 GO terms and KEGG pathways were shown in Fig. 2A,B. GO enrichment mainly included the modulation of chemical synaptic transmission, regulation of trans-synaptic signaling, presynapse, and syntaxin binding. KEGG pathways mainly included Neuroactive ligand-receptor interaction, cell adhesion molecules, and mineral absorption. Detailed results of GO and KEGG analysis were shown in Supplementary Table S6–S7.

### Establishment of the prognostic age-related gene signature

Univariate analysis was performed to evaluate associations between 279 DEGs and OS in the training set. In the light of the selection criteria (*P* < 0.05), 2 DEGs were demonstrated to be associated with the OS of CRC patients (Table 1). Subsequently, DLX2 and PCOLCE2 were selected to establish a prognostic model by multivariate Cox regression analysis (Table 2). The risk score was calculated with the following formula: risk score = [Expression level of DLX2 * (0.989191835)] + [Expression level of PCOLCE2 * (0.417405617)].

**Table 1:** Univariate Cox proportional hazard regression analysis of different genes.

Gene	HR	HR.95L	HR.H	*P* value
DLX2	2.666280196	1.413137639	5.03068483	0.002465452
PCOLCE2	1.524810809	1.067433191	2.178167237	0.020415715

**Table 2:** Multivariate Cox proportional hazard regression analysis.

Gene	Coef	HR	HR.95L	HR.95H	*P* value
DLX2	0.989191835	2.68906039	1.408967681	5.132158727	0.002702859
PCOLCE2	0.417405617	1.518018122	1.06348033	2.166828058	0.02150802

**Figure 3:**
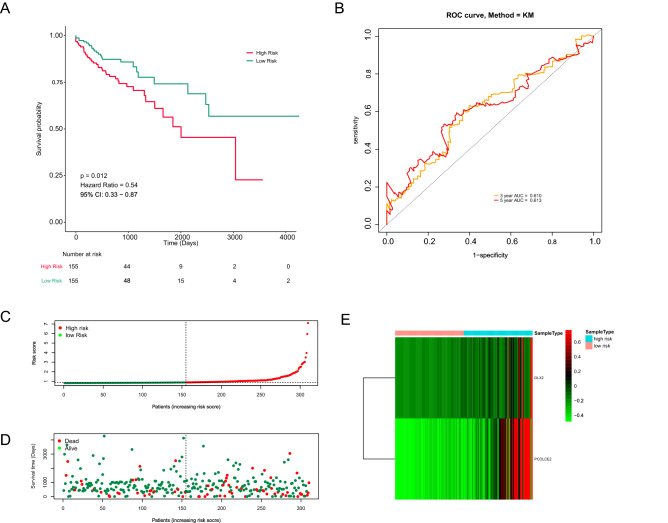
Combined DLX2 and PCOLCE2 genes in CRC overall survival prediction. (**A**) Kaplan–Meier survival curve of CRC patients between the low-risk group and the high-risk group. (**B**) 3- and 5-year ROC curve. (**C**, **D**) Risk score and survival status of the high-risk and low-risk groups. (**E**) The heat map of expression profile of the 2 prognostic genes. (software: pheatmap package in R, version number: 0.7.7, RUL link: https://cran.r-project.org/src/contrib/Archive/pheatmap/).

**Figure 4:**
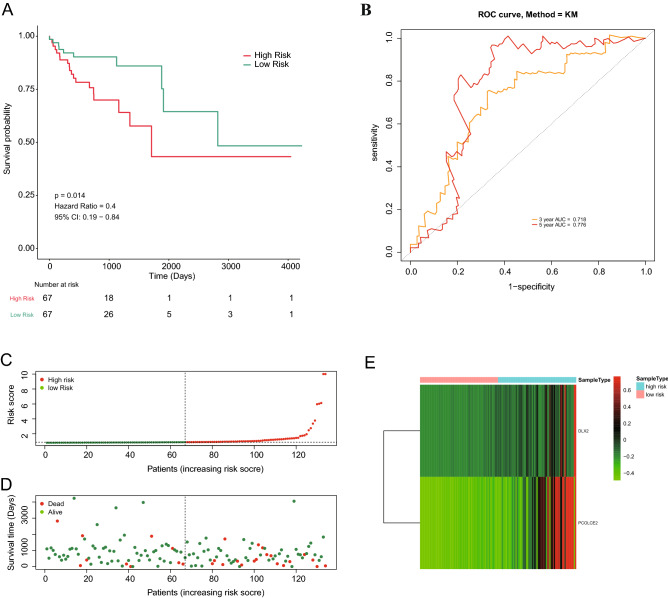
Validation of age related genes in CRC patients derived from TCGA dataset. (**A**) Kaplan–Meier survival curve of CRC patients between the low-risk group and the high-risk group. (**B**) 3- and 5-year ROC curve. (**C**, **D**) Risk score and survival status of the high-risk and low-risk groups. (**E**) The heat map of expression profile of the 2 prognostic genes. (software: pheatmap package in R, version number: 0.7.7, RUL link: https://cran.r-project.org/src/contrib/Archive/pheatmap/).

The CRC patients were divided into high- and low-risk groups according to the median risk score. K–M analysis showed that the survival probability in the high-risk group was significantly lower than that in the low-risk group (*P* = 0.012; Fig. 3A). As shown in Fig. 3B, the AUC values of 3- and 5-year ROC were 0.610 and 0.613, respectively. The survival state of each patient was shown in Fig. 3C,D. When the risk score increased, the number of deaths were also increased. The heatmap displayed expression profiles of 2 prognostic genes in high- and low-risk patients (Fig. 3E). The expression of two prognostic genes were all significantly up-regulated in the high-risk patients compared with the low-risk patients. We further verified the performance of the age-related prognostic gene in the validation set and achieved similar results. The median value of the risk score in the training set was used to divide the validation set into a high-risk group and a low-risk group. The survival probability of high-risk patients was significantly lower than that of low-risk patients (*P* = 0.014; Fig. 4A). The AUC of ROC at 3, and 5 years was 0.718 and 0.776 in the validation set, respectively (Fig. 4B). The risk score and survival state distribution were also visualized (Fig. 4C,D). The heatmap displayed the expression of five signature genes (Fig. 4E). On the whole, these results indicated the prognosis signature showed satisfactory performance in predicting the OS for CRC.

### Independent prognostic role of 2 gene signature

**Figure 5:**
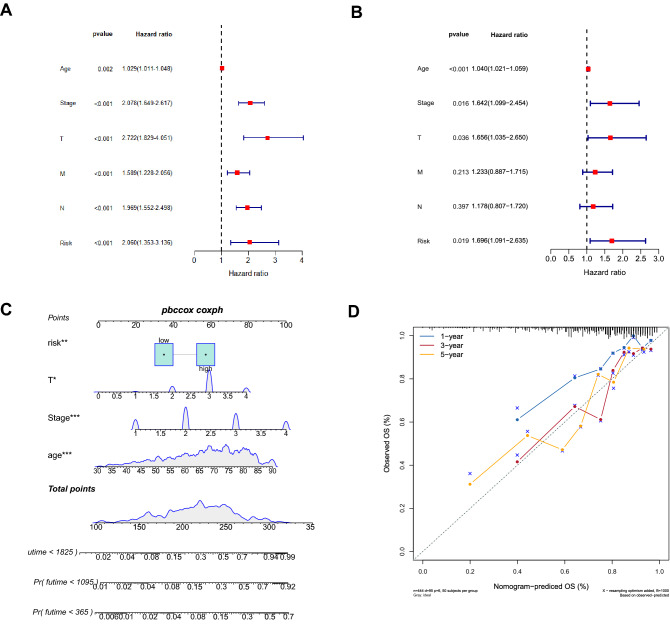
The relationship between clinicopathological factors and prognosis. (**A**) Univariate COX regression analysis forest map. (**B**) Multivariate COX regression analysis forest map. (**C**) A nomogram for predicting the probability of survival at 1, 3 and 5 years. (**D**) Nomogram calibration curve.

We performed univariate and multivariate Cox regression analyses to further investigate whether the risk was an independent prognostic factor for CRC patients. Univariate COX regression analysis indicated that the 5 clinicopathological characteristics (Age, Stage, T, N, M) and risk had statistical significance with OS of CRC patients (Fig. 5A). Multivariate COX regression analysis illustrated that risk was an independent prognostic factor of CRC patients (Fig. 5B). We further developed a nomogram to predict 1-, 3- and 5-year survival probability in CRC, according to the results from multivariate COX regression analysis (Fig. 5C). Moreover, calibration plots demonstrated that in comparison with the prognostic model, the nomogram had a similar performance (Fig. 5D).

### Gene set enrichment analysis with the 2 gene signature

**Figure 6:**
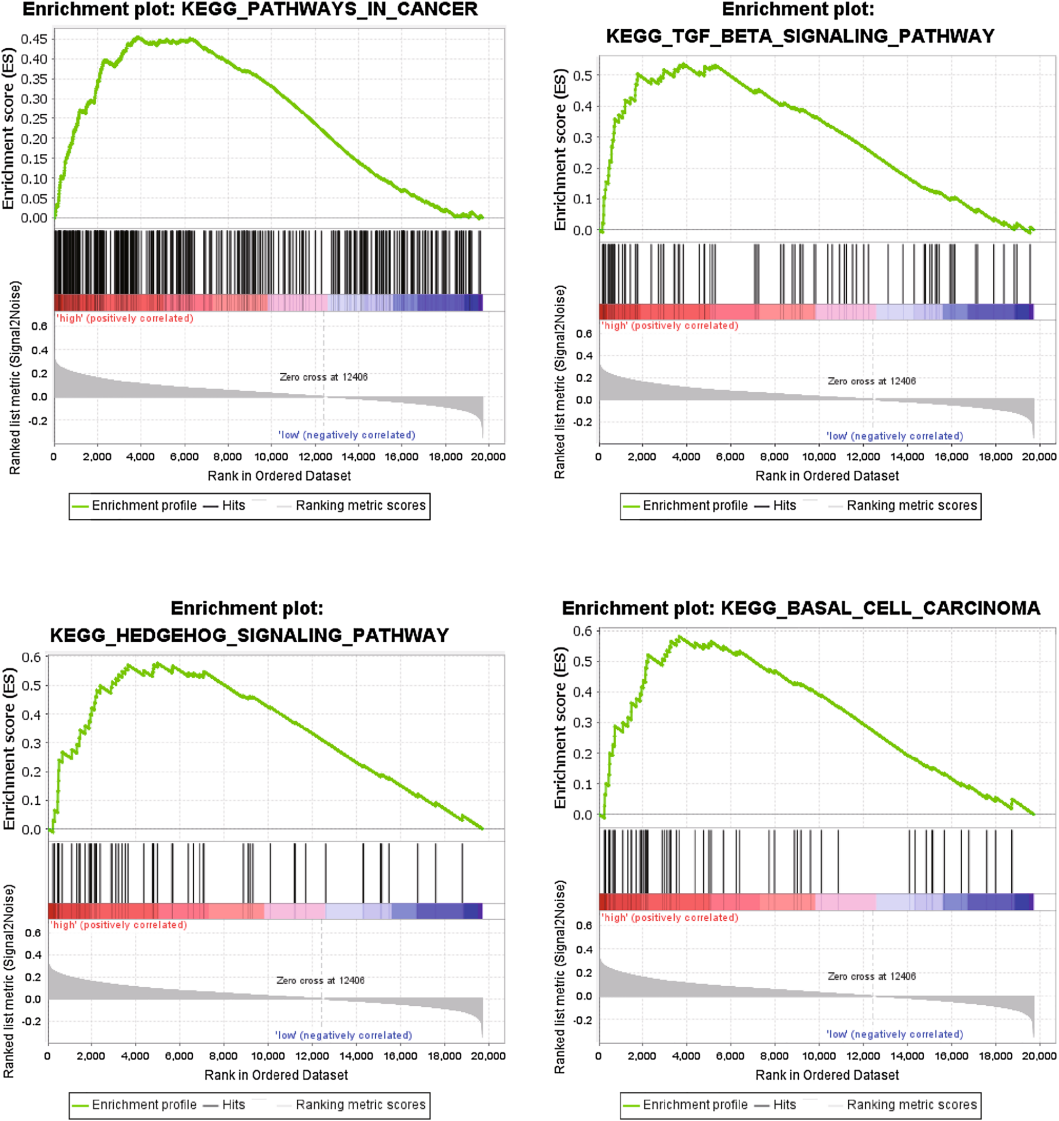
Gene set enrichment analysis with the 2 gene signature.

We performed GSEA of all genes between high- and low-risk groups in the whole TCGA database to explore the underlying biological mechanism of the gene signature. As is presented in Fig. 6 and Supplementary Table S8, KEGG pathways, such as ‘TGF BETA SIGNALING PATHWAY’, ‘GLYCOSAMINOGLYCAN BIOSYNTHESIS CHONDROITIN SULFATE’, ‘HEDGEHOG SIGNALING PATHWAY’, ‘BASAL CELL CARCINOMA’, and ‘PATHWAYS IN CANCER’ were significantly enriched in the high-risk group.

### Determination of the prognostic signature-related module by WGCNA

**Figure 7:**
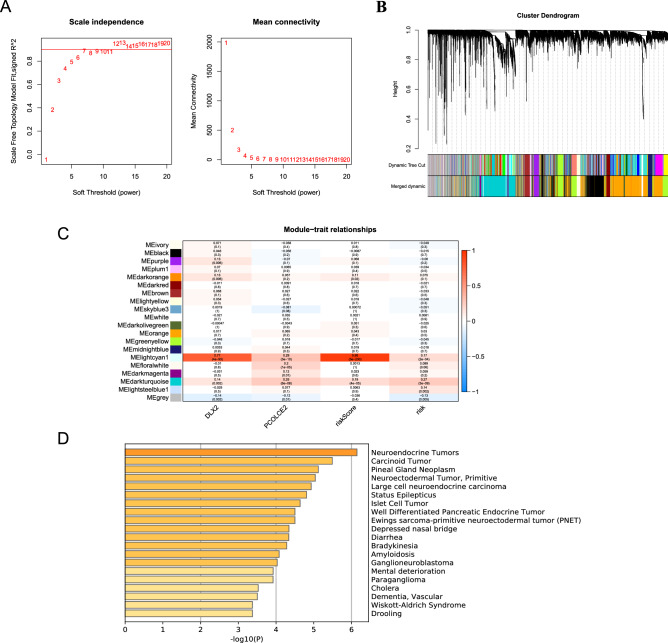
Weighted gene co-expression network analysis. (**A**) Soft-thresholding filtering. (**B**) Module screening, 20 modules have been identified. (**C**) The relationship between the 20 modules and the clinical traits. The numbers in rectangles indicate the correlation coefficient and the numbers in brackets indicate the *P* value. (**D**) Bar graph of enriched pathway of 43 genes in MElightcyan1 module.

Moreover, we also focus on the genes related to prognostic signature through constructed WGCNA co-expression network. By setting a soft-thresholding power of 7 (scale-free R^2^ = 0.9), we eventually identified 20 modules (Fig. 7A,B). Then, we performed a correlation analysis between 20 modules and clinical traits (DLX2 and PCOLCE2). As shown in Fig. 7C, the MElightcyan1 module had the highest Pearson coefficient with DLX2 (MElightcyan1, Cor = 0.77, *P* < 0.00001). The MElightcyan1 module contained a total of 43 genes, as shown in Supplementary Table S9. To further understand the biological functions of the 43 genes from the MElightcyan1 module, the Metascape website was adopted to carry out functional annotations of the genes. The results indicated that the 43 genes were associated with the cancer-related pathways, such as neuroendocrine tumor, carcinoid tumor, and islet cell tumor et al. (Fig. 7D).

## Discussion

Generally, the prevalence of cancer and the likelihood of a poor prognosis will increase with the advancing age^20^, but the exact mechanism of which is not clarified^21^. Although the increase in screening has contributed to the incidence decline in colorectal cancer, the incidence increased in younger patients and showed more aggressive tissue types^22,23^. In addition, early-onset colorectal cancer is associated with strong inherited predisposition and high incidence, and are more likely to develop metastatic disease during the course of the disease^24,25^. These observations indicate that age-related changes in colorectal cancer patients are complex and stimulate our interest in exploring age-related genes in colorectal cancer. This study analyzed the differentially expressed genes in elderly and young patients with colorectal cancer, and constructed a survival risk score model to explore the effects of age-related biomarkers on the prognosis of patients with colorectal cancer.

In this study, 279 differentially expressed age-related genes were screened. These include genes such as SYT4, HPSE2 and KCNB1, which have been reported to be associated with the development and potential treatment of colorectal cancer^26–28^. GO and KEGG enrichment analyses revealed the main pathways are neuroactive ligand–receptor interaction and cell adhesion molecules. SYT4 with strong protein–protein interactions in our study was reported to be involved in retrograde signaling along with cell adhesion complexes, which may be closely related to SYT4 regulating retrograde signaling and synaptic growth through shared and parallel signaling pathways^29^. Various cell adhesion molecules such as CEACAM-1 and CD44v6 are closely related to the invasion and metastasis of colorectal cancer^30–34^. Neuroactive ligand-receptor interactions have been shown to be associated with other gastrointestinal cancer^35^. Extensive research shows that chronic stress promotes the occurrence and progression of cancer. A recent study reported that the enrichment pathway of neuroactive ligand-receptor showed significant differences under different chronic stress conditions^36,37^. Stress has a negative impact on the neuroendocrine system and sympathetic nervous system. The threat of stress not only activates the sympathetic nervous system and increases catecholamine levels locally or systemically, but also increases inflammation and promotes tumor angiogenesis^38,39^. More and more studies have shown that oxidative stress is an important factor in the pathogenesis of colorectal cancer, these findings indicate that the exact functions and roles of neuroactive ligand-receptor in colorectal cancer are worthy of further exploration^40,41^.

After verification, we found that PCOLCE2 and DLX2 were an independent prognostic factors for CRC. PCOLCE2 encodes a functional procollagen c-protease enhancer, which is combined with five other genes to predict the risk of death in head and neck squamous cell carcinoma^42^. Our results are similar to previous findings, Chen et al. constructed a prognostic gene signature consisting of 9 genes including PCOLCE2 with a good prediction of overall survival in CRC patients^43^. Previous study reported mutations in PCOLCE2 in patients with rectal cancer, but the specific mechanism of PCOLCE2 in CRC is less known^44,45^. Recent studies found PCOLCE2 has been identified as the central gene of endometrial cancer progression, and low expression of PCOLCE2 is associated with longer overall survival duration which is consistent with our findings^46^. DLX2 is a member of distal-less homeobox gene family and has been confirmed to be involved in metabolic stresses occurring in solid tumors^47^. It has been reported to be associated with distant metastasis of colon adenocarcinoma and has been screened as a prognostic gene^48^. High DLX-2 expression is an adverse factor for cancer prognosis, degree of tumor differentiation, histological grade, and metastasis in a variety of cancers^49^. Regulating necrosis induced by metabolic stress and promoting the expression of markers of epithelial–mesenchymal transition are possible ways for DLX-2 to participate in tumor progression^47,50^. Previous studies examining the expression of DLX-2 in human tumor tissues have shown that DLX-2 mRNA levels are higher in some tumors compared to normal tissues, and DLX-2 expression is higher in tumor tissues than in matched non-tumorigenic tissues and stromal cells around cancer cells in colon cancers^47^.

GSEA analysis showed that high-risk samples were mainly enriched in cancer related pathways, such as TGF-β signaling pathway and hedgehog signaling pathway. TGF-β signaling pathway is one of the most important cellular pathways and has been reported to be involved in the initiation and maintenance of epithelial-to-mesenchymal transition in several malignant tumors^51^. Consistent with previous studies, activation of TGF-β signaling pathway plays a key role in the occurrence and development of colorectal cancer, and most colorectal cancers show mutation inactivation of TGF-β pathway^52^. Promoting IL-22 production in Th17 cells through AhR induction and PI3K signal transduction by TGF-β and loss of SMAD4 are possible underlying mechanisms^53,54^. It is feasible to regulate CXCR4 and its related targets to inhibit the growth and metastasis of colorectal cancer by inhibiting TGF-β signal, which further suggests the clinical significance of the pathway enrichment results in this study^55^. Hedgehog signaling is important for somatic development, deregulation of hedgehog signaling is closely related to tumor growth and developmental defects^56^. The existing research on the exact involvement of hedgehog pathway in the growth and metastasis of colorectal cancer is still very limited, although it has been reported to be associated with several cancers, its role in colorectal cancer is somewhat controversial^57^. Researchers have found that hedgehog signaling downstream cascade Gli family transcription factors play an important role in promoting colorectal cancer cell proliferation and tumor growth, while the exact regulatory mechanism is poorly understood^58,59^.

This study screened 43 genes from the MElightcyan 1 module by WGCNA, and further construction of the PPI network revealed the two core genes of CHGA and SYP. The research of Zhang et al. also supports our conclusions and verifies the diagnostic value of CHGA in colorectal cancer^60^. The synaptophysin protein encoded by the SYP gene is highly related to a variety of cancers such as hepatoblastoma and central neurocytoma^61^.

We constructed a two-gene risk signature based on age using the clinical information of colorectal cancer patients in the TCGA database to predict the prognosis of colorectal cancer patients. Univariate, multivariate Cox regression, and survival curve analysis all show that DLX2 and PCOLCE2 are significantly related to patient survival. Combined with clinical information (such as age, TNM), we also constructed a nomogram to predict the survival rate of patients. Our research has certain limitations. This predictive feature requires further clinical and experimental verification. We are also aware that although the effectiveness of the model has been verified, further research is needed to explore more biomarkers to improve the accuracy of model differentiation and improve clinical practice reference.

## Conclusion

DLX2 and PCOLCE2 are potential tumor markers of early-onset colorectal cancer. The high expression of DLX2 and PCOLCE2 may influence and participate in the occurrence of colorectal cancer, and lead to the occurrence of early-onset colorectal cancer. The results of this study provide an important reference for the prognosis of early-onset colorectal cancer. The relevant findings and conclusions obtained in this study still need further mechanism exploration and molecular verification in the future.

## Supplementary Information

Supplementary Table S1.

Supplementary Table S2.

Supplementary Table S3.

Supplementary Table S4.

Supplementary Table S5.

Supplementary Table S6.

Supplementary Table S7.

Supplementary Table S8.

Supplementary Table S9.

## Data Availability

The data used for analysis may be obtained in the supplementary information or from the authors upon reasonable request.
